# The supernumerary B chromosome of maize: drive and genomic conflict

**DOI:** 10.1098/rsob.210197

**Published:** 2021-11-03

**Authors:** James A. Birchler, Hua Yang

**Affiliations:** Division of Biological Sciences, University of Missouri, Columbia, MO 65211, USA

**Keywords:** mitotic drive, preferential fertilization, recombination, chromosomal knobs, genomic conflict, engineered minichromosomes

## Abstract

The supernumerary B chromosome of maize is dispensable, containing no vital genes, and thus is variable in number and presence in lines of maize. In order to be maintained in populations, it has a drive mechanism consisting of nondisjunction at the pollen mitosis that produces the two sperm cells, and then the sperm with the two B chromosomes has a preference for fertilizing the egg as opposed to the central cell in the process of double fertilization. The sequence of the B chromosome coupled with B chromosomal aberrations has localized features involved with nondisjunction and preferential fertilization, which are present at the centromeric region. The predicted genes from the sequence have paralogues dispersed across all A chromosomes and have widely different divergence times suggesting that they have transposed to the B chromosome over evolutionary time followed by degradation or have been co-opted for the selfish functions of the supernumerary chromosome.

## Graphical abstract

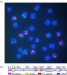

## 1. Introduction

Thousands of plants and animals have extra dispensable chromosomes called supernumerary or B chromosomes [1]. They typically do not associate with the normal set during meiosis and are of variable number between members of a population. It is assumed that they possess properties to maintain themselves despite being nonvital, although this has only been investigated in a few examples, which do indeed have drive mechanisms. The drive mechanisms of different B chromosomes are varied and can operate pre-meiotically, meiotically or post-meiotically, but all place more copies of themselves into the next generation than present in the previous generation. Thus, while B chromosomes are not needed, they maintain themselves in populations by these non-Mendelian mechanisms.

**Figure 1.**
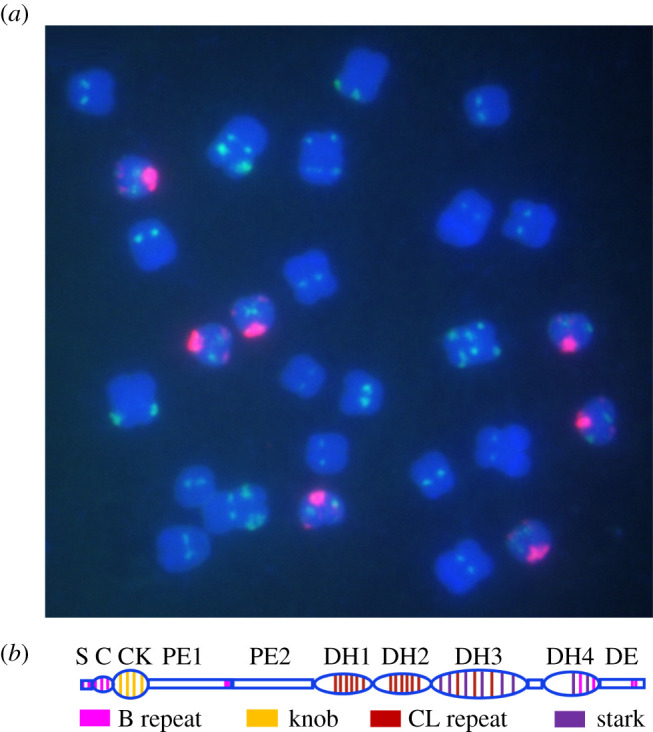
The maize B chromosome. (*a*) Mitotic metaphase spread of a line with seven B chromosomes (magenta). The magenta signal identifies the ZmBs (B specific repeat) in the centromere and short arm with a minor representative at the distal tip of the B long arm. Green signal identifies two chromosomal features, namely the CentC centromeric satellite and TAG microsatellite clusters. The green signal on the B chromosome represents CentC clusters, which have no centromere activity [26]. DAPI stains the chromosomes (blue). (*b*) Schematic view of the acrocentric maize B chromosome at pachynema of meiosis. The chromosome is divided into the B short arm (S), B centromere (C), centromeric knob (CK), proximal euchromatin (PE1-2), four blocks of distal heterochromatin (DH1-4) and the distal euchromatin (DE). Four representative repeats on the B chromosome including the B-specific repeat ZmBs, knob-180, CL-repeat and Stark repeat cluster are colour coded along with the length of the chromosome. Knob is present in the centromeric knob region. CL repeat is present at DH1, 2 and 3. The Stark repeat is present in most of DH3 and the distal portion of DH4.

**Figure 2.**
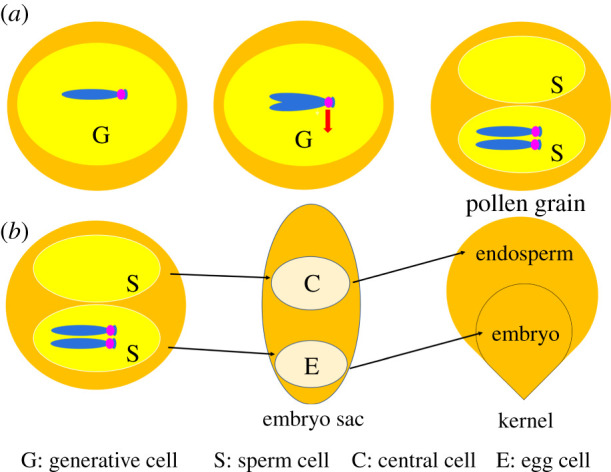
Components of the B drive mechanism. (*a*) Diagram of nondisjunction of the B chromosome in maize. The B chromosome (blue with a magenta centromere) is shown in the generative nucleus (G) after the first pollen mitosis. After replication, the two chromatids proceed to the same pole (red arrow) at the second pollen mitosis in the vast majority of divisions. Thus, most mature pollen grains contain two sperm cells (S) with only one containing the B chromosomes. (*b*) Depiction of preferential fertilization. For most lines of maize, the sperm with the two B chromosomes will preferentially fertilize the egg (E), as compared with the central cell (C) in the process of double fertilization. The fertilized egg develops into the next generation embryo and the fertilized central cell develops into the endosperm.

One of the most thoroughly studied B chromosomes is the one present in maize [2,3]. The supernumerary B chromosome of maize (figure 1) has several properties to perpetuate itself [2,3]. In most lines, the presence of the B is not detrimental to plant growth or development unless at high copy numbers beyond about 15 [4]. The B chromosome has a drive mechanism consisting of nondisjunction at the second pollen mitosis [5–7], which produces the two sperm cells, and then the B containing sperm preferentially fertilizes the egg in the process of double fertilization [8] (figure 2). The B chromosome modulates gene expression across the genome [9,10] and increases the rate of meiotic recombination on all chromosomes, particularly in heterochromatic regions and especially in male meiosis [11–18]. This process would foster the transmission of the B chromosome itself by insuring recombination in this largely heterochromatic chromosome, which would aid its own meiotic transmission [19]. The B chromosome has also evolved a mechanism to prevent its loss as a univalent in meiosis [20–22]. The nondisjunction mechanism requires two components: the centromere of the B [23], which has an additional specific repeat [24–26], and at least two trans-acting factors present at different sites on the chromosome [5,27–29]. The trans-acting factors are thought to delay further the replication of heterochromatin at the second pollen mitosis beyond its usual late duplication [30]. Thus, the B chromosome has manipulated cellular processes to ensure proper segregation in male meiosis by increasing recombination in heterochromatin, by ensuring its transmission if it is in a univalent state, by delaying replication of the centromere at the one mitosis that produces the two sperm and by mediating fertilization of the egg by the B containing sperm.

## 2. B-A translocations: genetic tools for maize

**Figure 3.**
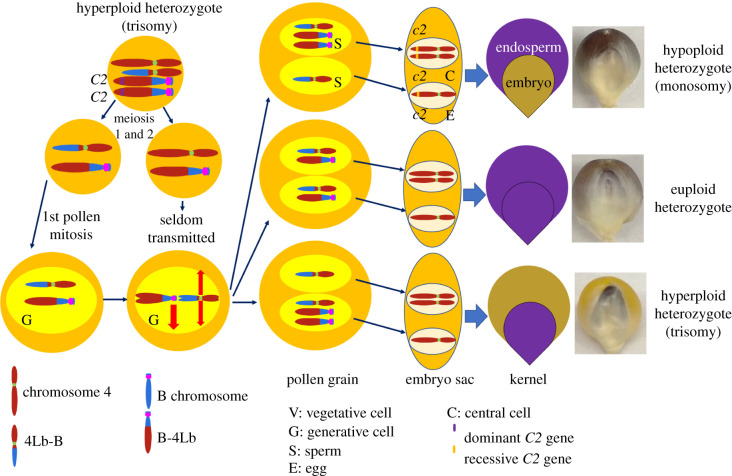
Diagram of the behaviour of a B-A translocation using TB-4Lb as an example. Hyperploid heterozygote (trisomy; 4 4Lb-B B-4Lb B-4Lb) containing a dominant *C*2 anthocyanin gene on the B-4Lb was crossed to a female *c*2 tester, which has no colour (yellow) in the kernel due to the blockage of anthocyanin pathway by the *c*2 mutation located on 4L. During meiosis, the TB-4Lb trisomy will produce two kinds of microspores, 4 B-4Lb and 4Lb-B B-4Lb. The 4 B-4Lb pollen have two copies of 4L and cannot compete with balanced 4Lb-B B-4Lb during pollen tube growth leading to only the latter being transmitted. During the second pollen mitosis, the nondisjunction of B-4Lb produces one sperm cell with two copies of B-4Lb and the other has zero copies of B-4Lb. Both sperm cells have the 4Lb-B chromosome. After double fertilization, the kernel would show coloured (purple) embryo and colourless endosperm (trisomy), or coloured endosperm and colourless embryo (monosomy). If the B-4Lb disjoins normally (right middle row), the kernel would show full colour (purple embryo and purple endosperm, euploid heterozygote). The copy number of 4L in monosomy, euploid and trisomy should be 1, 2 and 3 copies, respectively. The B portion is shown as blue with a magenta centromere and chromosome 4 is shown as dark red with a green centromere.

The property of the B chromosome to undergo nondisjunction at the very mitosis that produced the two maize sperm cells has been capitalized upon by maize researchers. Many features of the B chromosome have been deciphered using B-A translocations (figure 3), which add various genetic markers to the chromosome. A large collection of translocations between the B chromosome and A chromosomes has been induced [31,32]. Distal portions of A chromosome arms are joined with the B centromere. Thus, the A portion is also changed in dosage in the sperm and will lead to zygotes with different numbers of the respective chromosome arm. Of particular note and an exceptional advantage to maize is that heterozygous deficiencies can be produced because the nondisjunction occurs at the mitosis that produces the male gametes. Thus, extra or missing chromosome arms can be present in the male gametes at the endpoint of the haploid gametophyte generation that typically would abort if a large deletion were present. Because nondisjunction is not always 100%, some balanced gametes are also produced to generate a dosage series of 1, 2 and 3 copies in the resulting zygotes. B-A translocations have been used for uncovering recessive mutations [31], for numerous types of dosage studies [33–40], for understanding centromere structure [23,25,26,41–44], for examining the nature of the breakage–fusion–bridge (BFB) cycle [45,46], for the discovery of parental imprinting [47] as well as contributing to understanding the B chromosome itself.

## 3. Structure of the B chromosome

The B chromosome is nearly telocentric consisting mainly of a long arm and a minute short arm. It is highly heterochromatic. Beyond the centromere, it has a proximal euchromatic portion, followed by several large blocks of heterochromatin and terminating the long arm with a short euchromatic region (figure 1). The B chromosome contains a number of B specific repeats. One of these is located in and around the centromere with minor representations near the distal end of the chromosome [24]. The Stark repeat is present in two blocks of heterochromatin [48,49] and the CL repeat is present at several sites along the chromosome, primarily in the heterochromatic blocks [50–54]. The B specific repeat is related on the sequence level to knob heterochromatin present in the normal A chromosomes [24,55]. Knobs are repeated units at high copy number that stain deeply with chromatin stains. There are two major types in maize. The 180 bp knob repeat was the first recognized [56]. As its name implies, it consists of tandem repeats of 180 base pairs. These are typically present at interstitial sites on maize chromosomes [57,58], although a minor representation is present at the ends of all chromosomes [59], which is potentially the progenitor position given this is the place where they reside in relatives of maize such as *Zea luxurians* and *Tripsacum* species [60]. The second type of knob repeat is the TR1 repeat that is 350 bp in unit length but is related on the sequence level to the 180 bp repeat [61]. A 180 bp knob is present on the B chromosome in close proximity to the centromere.

As noted, the B specific centromeric repeat is related to the knob180 and TR1 heterochromatin repeats [24,55], especially to a portion of the knob sequence of highest evolutionary conservation [62]. Also, Cent4 is a repeat cluster found in all maize lines near the centromere of chromosome 4 [60] that is similar to the B repeat, having been isolated based on that homology [63]. Cent4, however, does not undergo nondisjunction in the presence of B chromosomes so there must be some distinction between the B repeat and the Cent4 array.

## 4. Sequence of the B chromosome

A high-quality sequence of the maize B chromosome has been produced [64]. There are 758 predicted protein-encoding genes. Paralogues in the A chromosome have widely distinct divergence estimates and genomic locations, suggesting that these predicted B genes have transposed to the supernumerary chromosome over evolutionary time [64]. Core genes in the grass family show evidence of synteny but none can be recognized with the B chromosome [64]. Synteny would be predicted whether the origin of the B chromosome occurred within a species or as detritus from a wide cross between species. However, whatever was the ultimate progenitor, the sequences have degenerated beyond recognition. This result suggests that the drive mechanism has propelled the chromosome through millions of years of evolution as a selfish entity. The maize B chromosome is a natural laboratory for transposed duplicate genes that are freed of purifying selection and linkage drag to evolve various selfish functions co-opted from normal. The maize B chromosome has recently been shown to contain active genes [9,10,49,64] and be capable of impacting the expression of genes from the A chromosomes [9,10].

An extensive collection of B centromere misdivision derivatives and other breaks in the surrounding region [44,65,66] made it possible to assemble 543 kb of the B centromere and order the scaffolds in relation to the chromosome, including the knob adjacent to the centromere. This length is close to a fiberFISH estimate of the core region of the B centromere [25]. The B chromosome centromere is very similar in repeat composition and organization to those on the A chromosomes with the exception of the interspersion of the B specific repeat throughout and flanking the core region.

## 5. Cis factors for nondisjunction

The B chromosome undergoes nondisjunction mainly at the second pollen mitosis, but there is also a low frequency of nondisjunction that occurs at the first pollen mitosis [5,7]. Further, there is a very low level of nondisjunction in the endosperm [41,67] and in the tapetum [68]. With copy numbers starting at about 6, and accelerating with increasing numbers, there is evidence for nondisjunction in sporophytic root tip cells [69]. The high copy number of the B chromosome appears to foster the nondisjunction property in a tissue where it does not otherwise occur at a detectable frequency.

Multiple investigators have examined replication of chromosomes in root tips and found that the 180 bp knobs were late replicating, as was the centromeric region of the B chromosome [30,70–72]. There is a knob near the B centromere, so Pryor *et al.* [30] postulated that this knob mediated the nondisjunction. This idea creates a conundrum because the knobs on A chromosomes in most backgrounds do not cause nondisjunction or chromosomal breakage. It was not until years later that the B centromere-specific repeat was discovered [24]. It surrounds and is interspersed in the centromere, and also surrounds the centromere-proximal knob [26]. Minichromosomes derived from the full B chromosome that are missing the knob are still capable of undergoing nondisjunction in the presence of the trans-acting factor containing portions of the B [73]. Thus, while the centromere proximal knob might contribute to nondisjunction, it is not solely responsible and the B specific repeat is implicated [64]. Indeed, the B specific repeat is more closely related to the TR1 heterochromatic knob repeat [55]. Wear *et al.* [72] found that the TR1 repeat was the most enriched in the late replicating sequences, despite its underrepresentation compared to the 180 bp knob [60], suggesting that it is the last or one of the last types of sequences replicated in maize S phase. Together the results imply that the B specific repeat region is very late replicating.

The activity of the B centromere is not needed for the B repeat to mediate nondisjunction. Han *et al.* [66] recovered an inactive B centromere present on the tip of the short arm of chromosome 9 (9S). It is attached to the normal chromosome 9 centromere so it is inherited through the life cycle and across generations. It is perfectly stable on its own. However, when full B chromosomes, which supply the trans-acting factors, are added to the genotype, this chromosome is induced to undergo nondisjunction at the second pollen mitosis or more frequently to break and set up a BFB cycle [74] in the subsequent endosperm development after fertilization. This is evidenced by a mosaic pattern for the anthocyanin, *C*1, marker gene on the 9S chromosome when crossed as a male to the recessive tester female stock. These results indicate that the hypothesized B repeat involvement in nondisjunction is independent of centromere function. Numerous lines of evidence point to a delayed or stalled replication of the B repeat cluster, and cytological visualization at the second pollen mitosis indicates that the B repeat array is the site of adherence of the inactive B centromere on separated chromosomes 9 [66].

A series of B chromosome centromere misdivision derivatives were found by Carlson [41]. These different derivatives have different rates of nondisjunction. The amount of the B specific centromere repeat was quantified in this series of chromosomes and the amount was correlated with the rate of nondisjunction [43,64]. These observations provide further evidence that the B specific repeat array is the sight mediating nondisjunction and that the rate of nondisjunction is related to the quantity present around the centromere.

## 6. Trans-acting factors for nondisjunction

One of the factors that operates in trans and that is required for nondisjunction (figure 3*a*) is present near the end of the long arm of the chromosome [5,27,29]. A second is present in the proximal euchromatin [28]. The most distal breakpoint of B-A translocations localized on the B chromosome sequence is that of TB-3Sb [64]. The B-3S chromosome contains most of the B chromosome except the very distal tip. This chromosome alone is incapable of nondisjunction at the second pollen mitosis [75]. Thus, the trans-acting factor #1 must reside in the small portion of the B translocated to the short arm of chromosome 3. This region of the sequence has 34 predicted protein-encoding genes [64].

## 7. Preferential fertilization

Preferential fertilization is the second aspect of the B chromosome drive mechanism and is determined by the female parent (figure 2*b*). In most maize lines, the preference for the egg versus the central cell is 2 : 1. However, some maize varieties do not allow preferential fertilization [76–80]. Multiple researchers [21,76–81] have found lines of maize in which there is a lack of preferential fertilization such that fertilization of the egg or the central cell by the B chromosome containing sperm is random rather than being skewed towards the egg, suggesting that there is a variation for this trait in maize populations. In Argentine maize varieties, there is evidence that this difference is controlled by a single gene [21]. Moreover, Carlson [77] has observed lines that, when used as a female parent for B-A translocations, show a reversal of preferential fertilization such that the polar nuclei are now favoured. Carlson [77] also found that adding several normal B chromosomes to the genotype would eliminate the reversal and change it to a random fertilization for the B-A translocation, when there is no difference between the two sperm for B centromere presence. Preferential fertilization of the egg in most lines is also eliminated by adding B chromosomes to the genotype to eliminate a difference between the two sperm [76]. At a low frequency in maize, two pollen tubes are involved with the fertilization of an embryo sac, a process called heterofertilization. Carlson [82] used this phenomenon to test whether preferential fertilization would follow the same frequency if both the egg and the polar nuclei were given a choice of sperm. The result was that preferential fertilization of the egg was at the usual skewed frequency in favour of the inclusion of the B centromere on a B-A translocation.

The most proximally broken B-A translocation when compared to the B sequence is TB-8Lc [64]. Its breakpoint is very near to the centromere [64]. Because this chromosome exhibits preferential fertilization, the difference between the two sperm cells after nondisjunction would essentially be the centromere. The implication is that the centromere or some associated protein, or modification of an associated protein, mediates preferential fertilization. Taken all together, it is probable that the B specific repeat cluster not only mediates nondisjunction at the second pollen mitosis but is also involved with preferential fertilization. At the very least, the two sites on the B chromosome that are involved with the drive mechanism are in very close proximity [64].

## 8. Effect on recombination

It has long been known that the presence of B chromosomes will increase the frequency of chiasmata visualized in meiosis across the genome [11]. As noted above, numerous subsequent studies showed that crossing over in the A chromosomes was enhanced by B chromosomes, particularly in centromeric heterochromatic regions where it is usually low. Rhoades [12] described a transposed segment of chromosome arm 3L into 9S, which normally has very low rates of crossing over, but showed a dramatic increase in the 3L region in the presence of B chromosomes.

Regions of the B chromosome have been assayed for the responsible segments involved in this modulation of genomic recombination. These studies have implicated the proximal euchromatin but there is also some evidence that the distal heterochromatin might have an effect as well [15,18]. The distal euchromatic tip has been definitively eliminated so this property of the B chromosome is distinct from the distal trans-acting factor required for nondisjunction [27]. Interestingly, the enhancement of recombination is typically greater on the male side than the female [13,14,17]. Thus, the B chromosome has modified the recombination process to favour its own crossing over, which has been shown to occur [83], in male meiosis to insure its faithful segregation and accurate distribution to all spores, which, of course, immediately precedes the other processes of its drive mechanism of nondisjunction and preferential fertilization. The B chromosome itself is highly heterochromatic so it has been postulated that this modulation of recombination is an evolutionary adaptation to help foster the orderly segregation of the supernumerary chromosome and hence its transmission to the next generation [19].

## 9. Stabilization of univalent transmission

**Figure 4.**
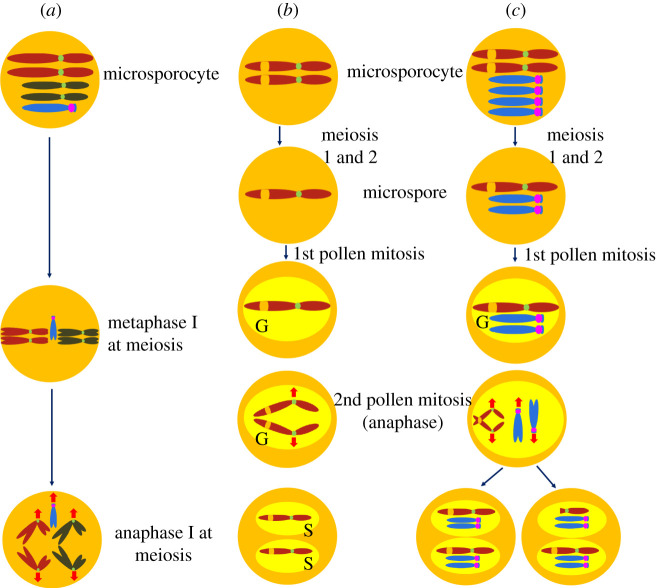
(*a*) Depiction of stabilization of univalent transmission of the B chromosome in meiosis. González-Sánchez *et al.* showed that a B univalent can be out of the plate and with the orientation to the poles at metaphase I of meiosis [22]. At meiotic anaphase I, the B chromosome proceeds to the poles ahead of the regular chromosomes, as shown by Carlson [20] and González-Sánchez [22]. These behaviours of the B chromosome are thought to facilitate the transmission of the B univalent. (*b*,*c*) Diagram of the high loss (HL) phenomenon induced by the presence of B chromosomes. From Rhoades & Dempsey's conclusions [88,89], in the HL line without the B chromosome, chromosome 3 with a large knob on 3L divides normally during pollen meiosis and mitosis. However, when a microsporocyte contains multiple B chromosomes such that the resulting microspores would contain two copies of the B chromosome, chromatids in the knob region on the 3L remain attached at the anaphase of the second pollen mitosis, whereas the centromere of chromosome 3 proceeds towards opposite poles. This process fractures 3L. The pollen grain will have one sperm cell with a truncated chromosome 3 and the normal chromosome 3 in the other sperm cell. In some cases, the knob region can divide before the fracture of 3L and both sperm cells have one copy of an intact chromosome 3. Note that the assortment of the nondisjoined B chromosomes is random, which means the B chromosomes could proceed towards the same or opposite poles (shown here).

An additional property of the B chromosome that fosters its transmission is its stabilization as a univalent in meiosis (figure 4*a*) [20,22]. Often, chromosomes as a singleton will lag in meiosis I anaphase and get lost, thus failing to be included in the products of meiosis. B chromosomes, because of their drive mechanism, can find themselves in odd numbers in meiosis. These multimers can take on bizarre associations [24,57,84], but usually the centromeres are in pairs and segregation proceeds. However, when univalent B chromosomes are present, they have been observed to proceed to the poles in meiotic anaphase I ahead of the regular chromosomes [20,22,57]. In some respects, this behaviour is analogous to the behaviour of neocentromere formation of knob heterochromatin in the presence of Abnormal chromosome 10 (Ab10) [85] but is clearly distinct [22]. Indeed, González-Sánchez *et al.* [22] presented evidence that the small distal array of B repeats were involved in this process despite it having no detectable CENH3 [26]. This observation might suggest that a poleward migration occurs that is independent of a normal kinetochore as occurs with Ab10 [86].

There are two systems of neocentromere formation associated with Ab10—one for knob180 and one for TR1 knobs [87]. They both involve neomorphic forms of kinesin motor proteins. In these cases, there is no involvement of CENH3, the histone variant typical of active centromeres, as a foundation for a kinetochore but nevertheless an attachment and movement occurs on the meiotic spindle. It is thus conceivable that the B chromosome has evolved a co-opted related mechanism in which univalents can attach to the spindle and proceed ahead of the normal chromosomes to the poles in meiotic anaphase I. The B specific repeat array has only a small portion of it that shows any association with CENH3 on a normal chromosome [26], but the whole array could act as a neocentromere in meiosis.

Carlson & Roseman [20] localized a B distal heterochromatic region required for the precocious progression of the B specific repeat array to the anaphase I poles of meiosis. If the centromere itself is subject to a novel meiotic progression, it would not result in meiotic drive as occurs with Ab10, which relies on recombination between centromeres and the sites of alternative sizes of knob heterochromatin for drive to occur. Instead, a similar process for the B centromere insures that univalents can succeed in passing through meiosis. The sequence of the B chromosome [64] indicates that it is not related at all to chromosome 10.

While it might seem counterintuitive to suggest that a centromere sequence would acquire neocentromere activity, there are data in the literature to suggest that is the case. Images of B chromosomes in meiosis have long shown that univalents in meiotic anaphase I progress to a pole before the normal chromosomes (e.g. [57]). Carlson & Roseman [20] studied this feature in detail and found that an intact centromeric region was necessary and that the distal heterochromatin appeared to control this activity. We now understand that the chromosomes that Carlson & Roseman analysed were reduced in the amount of centromeric B-specific repeat [26,43,44]. The B centromere alone does not exhibit this behaviour [85], supporting the claim for a trans-acting factor, which would be analogous to neocentromere formation by Ab10 [86,87].

## 10. Impact on the A chromosome integrity

Rhoades & Dempsey [88,89] discovered a line of maize (called High Loss (HL)) in which the presence of at least two B chromosomes in a microspore (3 or more per plant) would cause the heterochromatic knobs on the A chromosomes to break at the second pollen mitosis, the same mitosis at which the B chromosome centromere remains adhered during anaphase to produce nondisjunction (figure 4*b*,*c*). The line with B chromosomes also produced an unusual frequency of trisomies and triploids when used as a male. They noted that transmission of an extra chromosome through the male is unlikely and that triploid plants are highly sterile so they must arise anew. It seems that the HL characteristic in fact causes nondisjunction of A chromosomes at the second pollen mitosis in order to obtain such individuals in the progeny, although whether this is conditioned by the unreplicated knobs or A centromeric heterochromatin is unresolved. In an attempt to test whether the B chromosomes originally in this line were unique, they introgressed B chromosomes from other lines, which they found were also effective. Their results indicate that the effect occurs in the gametophyte generation but also that there is a single major gene difference between ‘HL’ and other lines harbouring B chromosomes, although there were also numerous modifiers that would affect the severity, as is true of almost any quantitative characteristic. In the HL line, the delay of replication of heterochromatin is so severe as to cause fracture of many chromosomes in the genome and apparent nondisjunction of normal chromosomes [88,89]. The B chromosome relies on this delay for its perpetuation but the normal genome can be severely damaged if it is too late.

## 11. Conflict between the B and A chromosomes

The B chromosome is widespread in Mexican teosintes, the wild maize relative, as are the major knob positions that have descended into maize [57]. However, multiple investigators have noted that in landraces of maize, there is a negative correlation between the presence of B chromosomes and of many knobs [90,91]. The combination of these studies suggests that teosinte and most maize lines have variants that prevent the HL syndrome from occurring. However, the landraces in which the B chromosomes are prevalent but not knobs might have variants that allow the HL delay of knob replication to occur.

## 12. Use of the B chromosome as a platform for engineered minichromosomes

Because of its dispensable nature, the B chromosome could serve as a platform to develop engineered minichromosomes. Centromeres in plants are epigenetically based [92] and thus do not rely on DNA sequence for function. Thus, the baker's yeast example of producing artificial chromosomes by assembling centromeres, telomeres and marker genes *in vitro* with reintroduction to cells will not work in plant species. To overcome this issue, telomere-mediated chromosomal truncation was used to remove the ends of chromosomes and simultaneously place genes at the tip [93]. The B chromosome of maize was an initial target although the procedure can be applied to other chromosomes [94]. The truncation event for the B chromosome removes the terminal trans-acting factor that is needed for nondisjunction, so the engineered minichromosomes disjoin normally at the second pollen mitosis. However, in the presence of full-sized B chromosomes that provide the trans-acting functions, the minichromosomes will undergo nondisjunction [73]. Various applications of B derived engineered minichromosomes have been described [95].

## 13. More to learn

The B chromosome is a mysterious chromosome. Genes that transpose to the B will degenerate unless they contribute in some way to the perpetuation of the chromosome in the multitude of ways described above. This seems to have occurred to great effect. The B chromosome uses a unique repetitive sequence in and around the centromere to cause nondisjunction at the one mitosis giving rise to the male gametes. There are trans-acting factors that mediate this effect on the repetitive array. How this occurs remains a mystery and is an interesting avenue for further study. Then, the sperm that has the nondisjoined chromosomes somehow preferentially joins with the egg as opposed to the central cell in the process of double fertilization—again by an unknown process. Still further, the B chromosome has manipulated the meiotic recombination process to foster crossing over in its heterochromatic structure and brought an increase in proximal regions across the genome. Increasing recombination between paired B chromosomes would foster faithful distribution into the resulting spores from meiosis and eventual transmission to the next generation. However, if the B chromosome is present as a univalent, it has also developed a mechanism that can foster its transmission in this state, apparently by a distinct mechanism from normal centromere functions. This amazing array of properties illustrates how selfish entities can acquire a multitude of characteristics to propel themselves into future generations when uncoupled from normal chromosomes and having no vital functions.

## Data accessibility

This article has no additional data.
